# Successful Treatment of Refractory Synovitis, Acne, Pustulosis, Hyperostosis, and Osteitis (SAPHO) Syndrome With Tofacitinib: A Case Report

**DOI:** 10.7759/cureus.66169

**Published:** 2024-08-05

**Authors:** Mustafa Alhayali

**Affiliations:** 1 Rheumatology, Center of Spine and Joint Diseases, Baghdad, IRQ

**Keywords:** acne, tofacitinib, jak inhibitor, refractory, sapho syndrome

## Abstract

Synovitis, acne, pustulosis, hyperostosis, and osteitis (SAPHO) syndrome is primarily manifested by persistent inflammation affecting the musculoskeletal system and the skin. The treatment of SAPHO syndrome remains a challenge. Tofacitinib is a Janus kinase (JAK) inhibitor that inhibits a range of cytokines. Here, we report a patient who had been diagnosed with SAPHO syndrome refractory to initial treatment and responded well to tofacitinib. An 18-year-old male was presented to our center with polyarthritis, associated with sternal and clavicular pain. There was a nine-month history of skin lesions affecting his chest and back and was diagnosed with a case of SAPHO syndrome. Nonsteroidal anti-inflammatory drugs, conventional disease-modifying antirheumatic agents, and biological drugs were unhelpful. After five weeks of starting tofacitinib at 5mg twice daily in combination with methotrexate, the patient reported significant improvement in dermatological and osteoarticular symptoms. JAK inhibitors, especially tofacitinib, can be a good choice for the treatment of SAPHO refractory to disease-modifying antirheumatic drugs (DMARDs) and tumor necrosis factor (TNF) inhibitors.

## Introduction

Synovitis, acne, pustulosis, hyperostosis, and osteitis (SAPHO) syndrome has been described as a syndrome in medical literature since 1987 [1]. It is primarily manifested by persistent inflammation affecting the musculoskeletal system and the skin [2]. SAPHO syndrome is a rare disease, with limited data available regarding its prevalence, which has been estimated as 1:10000 in White populations [3]. Despite the fact that prior studies showed that genetic predisposition, certain infections, and immune dysregulation are linked to the development of SAPHO syndrome, the exact etiology and pathogenesis remain incompletely explained [4]. Due to disease rarity, multisystemic involvement, high clinical diversity, and a lack of specific investigations, diagnosing and treating this disease is challenging.

Clinical features of SAPHO syndrome include synovitis, which is often inflammatory non-erosive arthritis, osteitis with focal bone inflammation and occasional swelling, hyperostosis, which is bone overgrowth and cortical thickening, and dermatological manifestations such as palmoplantar pustulosis and acne which are the most frequently reported skin manifestations [5]. Here, we report a patient who had been diagnosed with SAPHO syndrome refractory to initial treatment and responded well to tofacitinib.

## Case presentation

An 18-year-old male from Diyala, Iraq presented to our center with joint pain and swelling involving wrists, shoulders, and right knee for about three weeks before his initial visit. He also suffered from sternal and right clavicular pain which gradually became more severe. There was a nine-month history of skin lesions affecting his chest and back and was diagnosed with acne conglobata. The patient denied fever, inflammatory back or neck pain, and weight loss. His family history was significant as his older brother was diagnosed with ankylosing spondylitis and uveitis and is currently under treatment with adalimumab.

**Figure 1:**
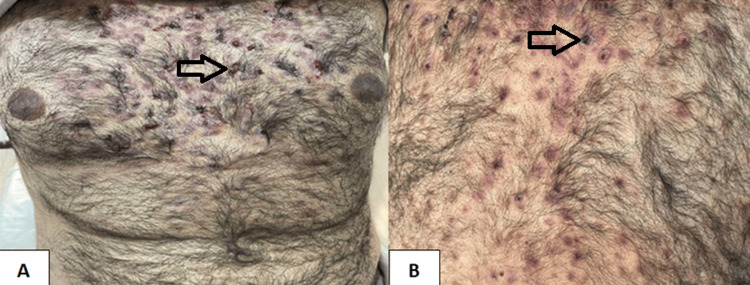
Acneiform lesions and pustules (arrows) affecting the anterior chest wall (A) and back (B).

Physical examination revealed arthritis of the aforementioned joints, tenderness over the sternum and right clavicle, and multiple acneiform lesions affecting the anterior chest wall and back (Figure 1). The rest of the physical examination was normal.

His initial workup showed an erythrocyte sedimentation rate (ESR) of 48 mm/hour (normal range: 0-20 mm/hour), a C-reactive protein (CRP) level of 86 mg/L (normal range: <10 mg/L), and a white blood cell count of 12 x 10^9/L (normal range: 4-10 x 10^9/L). The rest of the laboratories and radiological studies were not significant. Based on his clinical presentation, laboratory findings, and the opinion of the dermatologist, he was diagnosed with a case of SAPHO syndrome.

We started etoricoxib and doxycycline in full doses for three weeks without satisfactory results. Therefore, a decision to start oral methotrexate at a dose of 15 mg weekly in addition to etoricoxib was made. During the follow-up visit, the patient was still suffering from joint pain and stiffness in addition to chest wall pain and skin lesions. Anti-tumor necrosis factor (anti-TNF) treatment with subcutaneous adalimumab 40 mg every two weeks was given to the patient for another three months without significant improvement.

**Figure 2:**
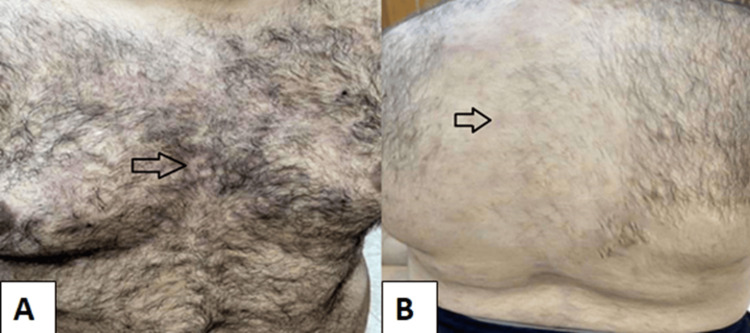
Resolution of skin lesions with residual erythema (A) and hyperpigmentation (B).

Lastly, we started Janus kinase (JAK) inhibitor tofacitinib at a dose of 5 mg twice daily in addition to oral methotrexate 15 mg weekly. After five weeks, the patient reported good improvement in both musculoskeletal and dermatological manifestations (Figure 2), with a decline in inflammatory markers (ESR was 23 mm/hour, CRP was 9.5 mg/L).

During the last follow-up visit, there was a complete resolution of articular symptoms and sustained improvement of skin manifestations and inflammatory markers (ESR was 18 mm/hour, CRP was 4.4 mg/L).

## Discussion

SAPHO syndrome is a chronic autoimmune disease that is manifested by raised levels of different inflammatory cytokines and neutrophil stimulation; among these changes, the upregulation of TNF-alpha, interleukin (IL)-8, IL-17, and IL-1b was recorded [6,7].

The treatment of SAPHO syndrome remains a challenge. Treatment mainly aims at relief of disease symptoms and prevention of exacerbations, which may affect patients’ quality of life. Currently, standardized treatment guidelines for SAPHO syndrome are lacking [7]. The treatment plan includes nonsteroidal anti-inflammatory drugs (NSAIDs), corticosteroids, disease-modifying antirheumatic drugs (DMARDs), bisphosphonates, and doxycycline based on case reports, and expert opinions [1,7]. NSAIDS and analgesia are usually given as the first-line treatment [7]. Our patient used etoricoxib for three weeks without noticeable improvement. The use of conventional synthetic DMARDs (csDMARDs) such as methotrexate, sulfasalazine, and azathioprine has shown inconsistent results [1,7]. In our case, methotrexate was ineffective. We did not choose a second csDMARD due to the severity of symptoms and axial (chest wall) involvement. Biological agents such as anti-IL-1 biologics [8] and IL-17 and 23 targeted therapies [9] have been described. However, these agents are not available in Iraq. Moreover, the use of anti-TNF drugs is supported by some case reports [10,11]. However, our patient did not have a significant response to the use of adalimumab. Fortunately, he responded dramatically to tofacitinib with a resolution of dermatological and osteoarticular manifestations.

Tofacitinib is a JAK 1 and 3 inhibitor that demonstrated its effectiveness in some rheumatic conditions [12]. By inhibiting JAK3, it blocks IL-2, IL-4, IL-6, IL-7, IL-9, IL-15, and IL-21; and by JAK1 inhibition, it blocks IL-6, type 1 interferon, and interferon-gamma [13]. Some common adverse effects of tofacitinib include cytopenia and infection [14], gastrointestinal perforation [15] and thrombosis [16]. These adverse effects were considered and monitored during the course of treatment.

To the best of our knowledge, there are few published case reports about using tofacitinib in the treatment of SAPHO syndrome. In 2020, a pilot study conducted by Li Y et al. at Peking Union Medical College Hospital [17], where 12 patients treated with tofacitinib showed significant improvements, like in our case. Recently, a case report was published by Yuan et al. about a patient diagnosed with SAPHO syndrome associated with ankylosing spondylitis which was successfully managed with tofacitinib [18]. Another case report was published by Yang et al. who used baricitinib, another JAK inhibitor in the treatment of SAPHO syndrome successfully [19].

Firinu et al. [7] explained that chest wall involvement, largely that of the sternum and clavicles, is common in SAPHO, which is similar to the presentation of our patient. In terms of spine involvement [20], the lesions affect the thoracic spine while sacroiliac inflammation can be seen and is frequently unilateral and affects mainly the iliac side of the sacroiliac joint, while in our patient, there was no spine or sacroiliac involvement.

## Conclusions

SAPHO syndrome is a chronic condition that affects the skin and the musculoskeletal system; it can be a difficult-to-treat condition with disabling effects on the patient's quality of life. There are no standardized treatment guidelines for SAPHO syndrome, and the management depends mainly on case reports and expert opinions.

Our findings suggest that JAK inhibitors, especially tofacitinib, can be a good choice for the treatment of SAPHO syndrome and other inflammatory diseases. The rapid and dramatic response of this patient to tofacitinib shows that JAK inhibitors offer a viable alternative to NSAIDs, csDMARDs, and TNF inhibitors, especially in resistant cases. Adverse effects of tofacitinib including cytopenia, infection, gastrointestinal perforation, and thrombosis should be addressed and followed up during the course of treatment. More future studies are needed to confirm the long-term efficacy and safety of tofacitinib in patients with SAPHO syndrome.
